# Supplementary material for: Effect of Group Mixing and Available Space on Performance, Feeding Behavior, and Fecal Microbiota Composition during the Growth Period of Pigs
Source: Animals (Basel). 2024 Sep 18;14(18):2704. doi: 10.3390/ani14182704 (PMC11428945; doi:10.3390/ani14182704)

Supplementary Figure S1. Relative abundance across time-points of (A) Phylum, (B) Genus

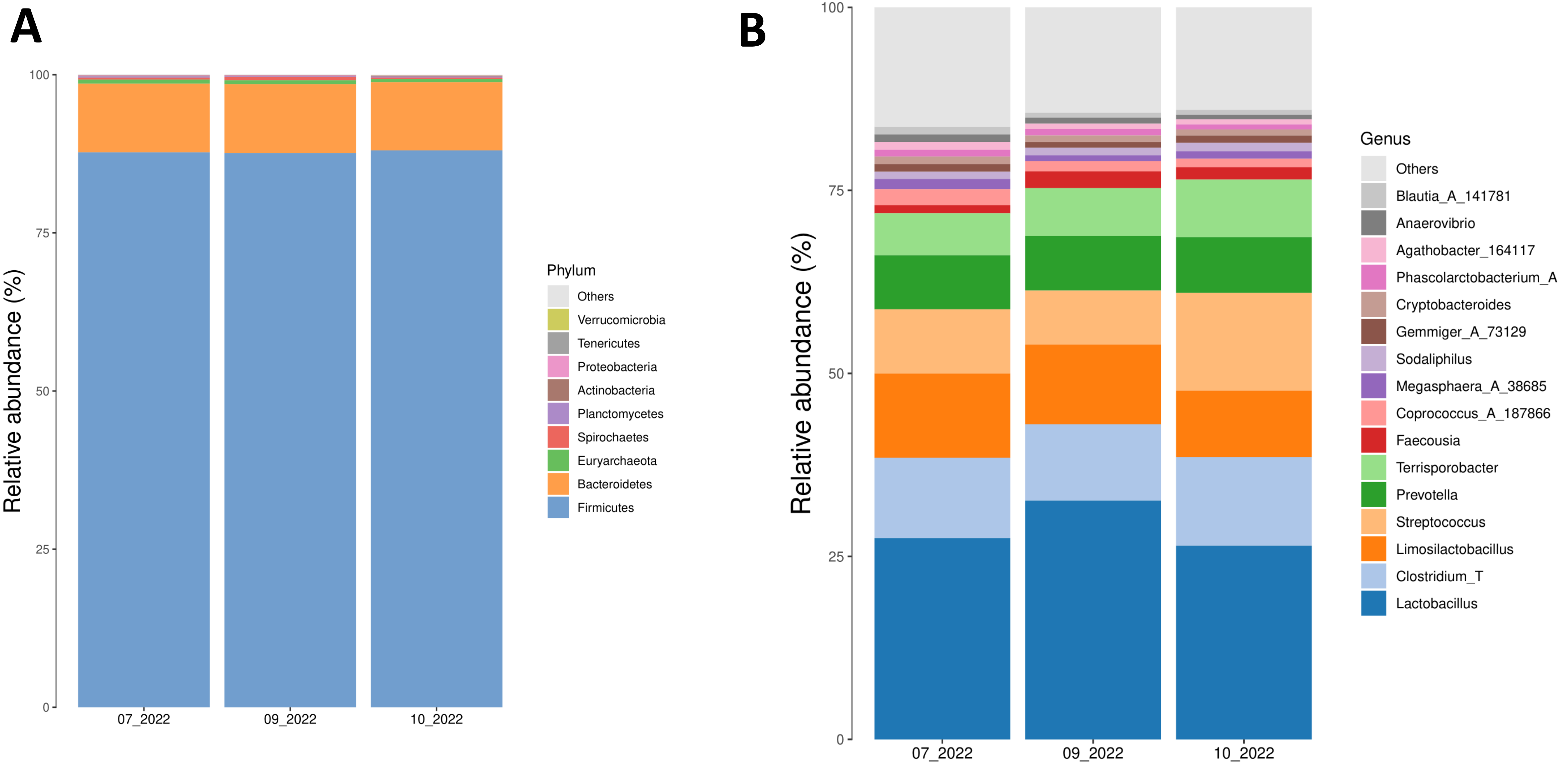

Supplementary Figure S2. Mean comparison of node degree between control and stress networks

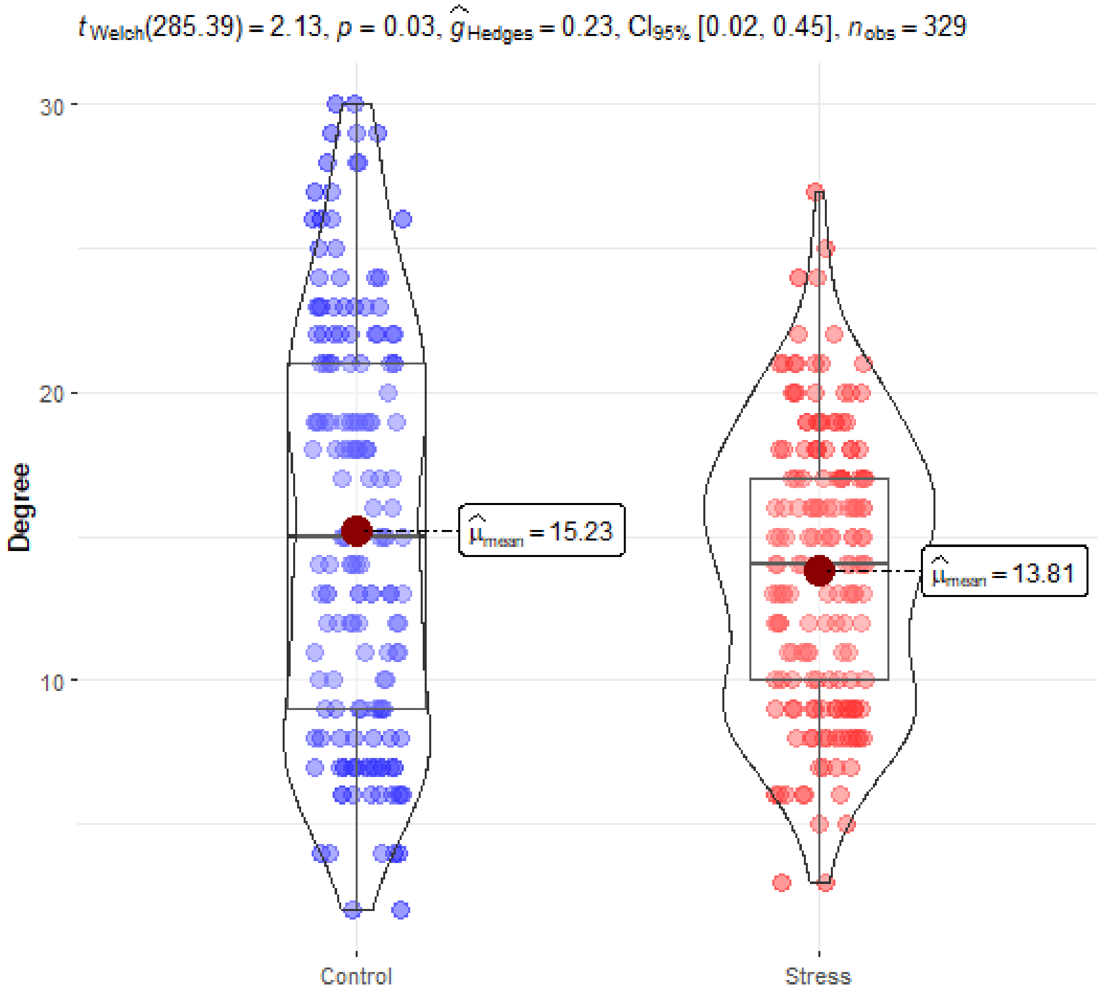

Supplementary Figure S3. Dynamic patterns of the clr-abundance of genera indicators of acuate stress

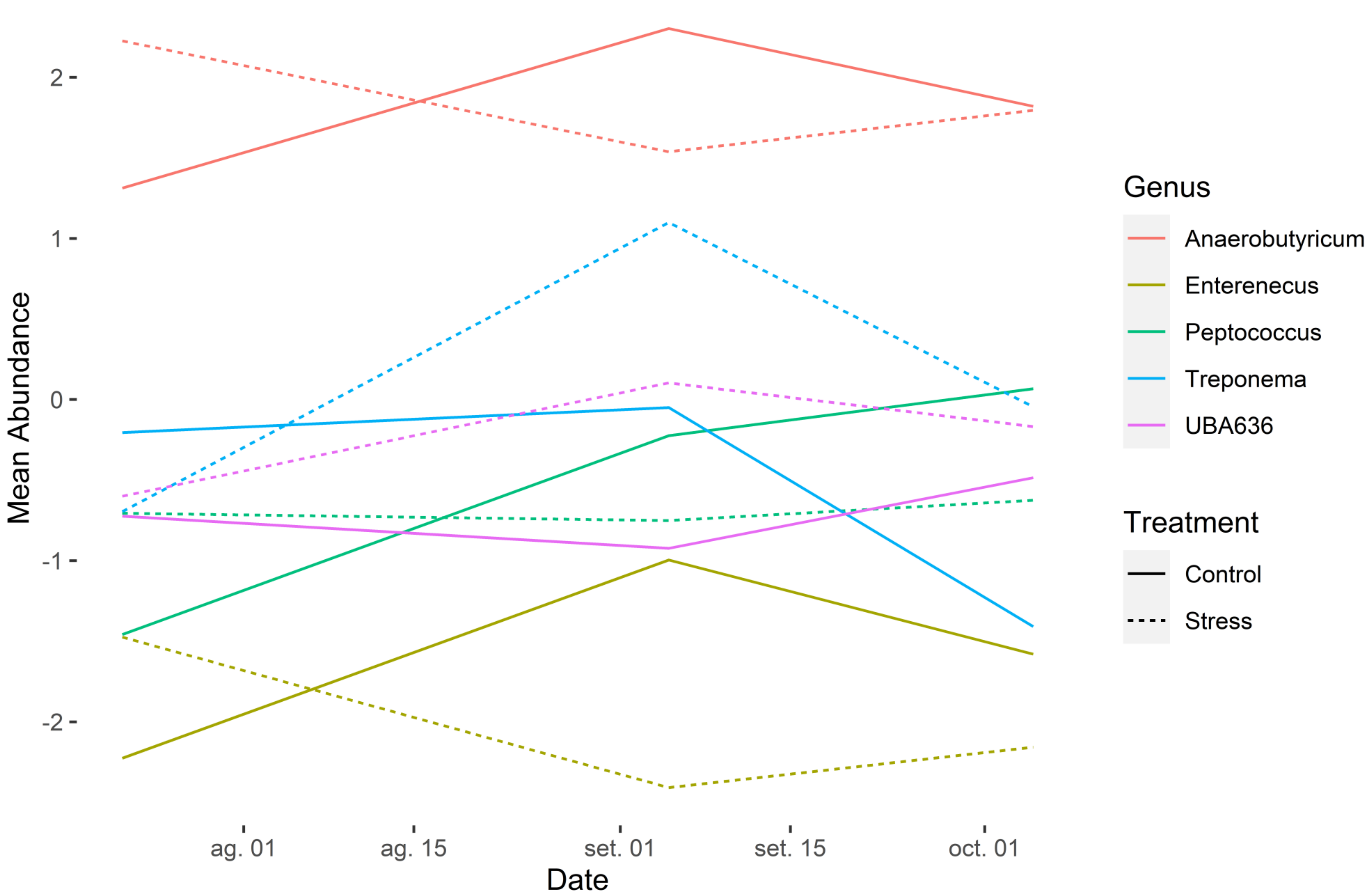

Supplement: Supplementary file 1 [file animals-14-02704-s001.zip › Supplementary_figures.pdf]
